# Dual Transcriptomic Profiling of Host and Microbiota during Health and Disease in Pediatric Asthma

**DOI:** 10.1371/journal.pone.0131819

**Published:** 2015-06-30

**Authors:** Marcos Pérez-Losada, Eduardo Castro-Nallar, Matthew L. Bendall, Robert J. Freishtat, Keith A. Crandall

**Affiliations:** 1 Computational Biology Institute, George Washington University, Ashburn, Virginia, United States of America; 2 Division of Emergency Medicine, Children’s National Medical Center, Washington, DC, United States of America; 3 CIBIO-InBIO, Centro de Investigação em Biodiversidade e Recursos Genéticos, Universidade do Porto, Campus Agrário de Vairão, Vairão, Portugal; 4 Universidad Andrés Bello, Center for Bioinformatics and Integrative Biology, Facultad de Ciencias Biológicas, Santiago, Chile; University of Illinois at Urbana-Champaign, UNITED STATES

## Abstract

**Background:**

High-throughput sequencing (HTS) analysis of microbial communities from the respiratory airways has heavily relied on the 16S rRNA gene. Given the intrinsic limitations of this approach, airway microbiome research has focused on assessing bacterial composition during health and disease, and its variation in relation to clinical and environmental factors, or other microbiomes. Consequently, very little effort has been dedicated to describing the functional characteristics of the airway microbiota and even less to explore the microbe-host interactions. Here we present a simultaneous assessment of microbiome and host functional diversity and host-microbe interactions from the same RNA-seq experiment, while accounting for variation in clinical metadata.

**Methods:**

Transcriptomic (host) and metatranscriptomic (microbiota) sequences from the nasal epithelium of 8 asthmatics and 6 healthy controls were separated *in silico* and mapped to available human and NCBI-NR protein reference databases. Human genes differentially expressed in asthmatics and controls were then used to infer upstream regulators involved in immune and inflammatory responses. Concomitantly, microbial genes were mapped to metabolic databases (COG, SEED, and KEGG) to infer microbial functions differentially expressed in asthmatics and controls. Finally, multivariate analysis was applied to find associations between microbiome characteristics and host upstream regulators while accounting for clinical variation.

**Results and Discussion:**

Our study showed significant differences in the metabolism of microbiomes from asthmatic and non-asthmatic children for up to 25% of the functional properties tested. Enrichment analysis of 499 differentially expressed host genes for inflammatory and immune responses revealed 43 upstream regulators differentially activated in asthma. Microbial adhesion (virulence) and Proteobacteria abundance were significantly associated with variation in the expression of the upstream regulator IL1A; suggesting that microbiome characteristics modulate host inflammatory and immune systems during asthma.

## Introduction

The application of novel, culture-independent techniques of high-throughput sequencing (HTS) is transforming our understanding of the role of microbes in respiratory illnesses such as asthma. HTS of specific microbial genes or whole genomes has first demonstrated that the pulmonary tract, historically considered sterile in health, contains diverse communities of microbes, i.e., the airway microbiome [1–4]. Since then, several studies have used HTS to estimate microbiome composition during health and disease (see reviews in [5, 6–9]) and revealed changes in the relative abundances of microbial groups (e.g., Proteobacteria, Bacteroidetes) and pathogenic taxa (e.g., *Moraxella*, *Haemophilus* or *Streptococcus* species) during asthma [10–12]–although some have challenged this statement [13]. Microbiome research has also shown associations between different bacterial community profiles, asthma phenotypes and specific clinical features (e.g., body mass index, neutrophil counts, antibiotic usage) [14]; and suggested that environmental factors (e.g., farms, pets, siblings, upbringing) or other microbiotas (i.e., gastrointestinal, oral) may also alter respiratory tract immune function in infancy and play a role in the development of asthma [5, 14].

But as with most respiratory illnesses, the study of the microbiome in asthma is still in its infancy; to our knowledge, no study has investigated the metabolic functions of the microbial communities residing in the airways, or to what extent the microbes that make up health- and asthma-associated communities (or other respiratory diseases) may also change their respective activities. Functional analyses of microbial communities may result even more informative and valuable than taxonomic characterization for understanding the role of microbes in health and disease [15, 16]. Microbiome functional analyses of illnesses such as periodontal and inflammatory bowel diseases, for example, have revealed major shifts in metabolic pathways related to disease pathogenesis in gut and oral microbiomes [17–20]. Considering that the genomic potential of the human oral and gut microbiomes are far greater than that of their host [21–23], and that previous studies have detected in the bronchial tree an average of 2,000 bacterial genomes per cm^2^ [3], one could expect that dysbioses in asthma may result not only from compositional changes in the airway microbiota, but also from shifts in fundamental microbial metabolic functions.

Similarly, although it is accepted that microbes contribute to mucosal inflammation in asthma, our immunological relation with the airway microbiota is still poorly understood [24]. New insights into the human’s innate sensing systems are beginning to delineate the mechanisms of host signaling and innate and specific immune response to microbes in the respiratory mucosa [24]; however, no study has assessed to what extent changes in the microbial communities constituting the microbiome contribute to mucosal inflammation or modulate host immune response during asthma. Such studies are beginning to emerge in other areas and have shown how shifts in the composition and function of the gut microbiota contribute to pediatric Crohn’s disease pathogenesis and inflammatory bowel disease [15, 19] or affect the expression of host genes associated with the innate immune system in epithelial cells [20].

Asthma microbiome research has mainly relied on the 16S rRNA gene. 16S is the gold standard for bacterial taxonomic profiling, but cannot be used to characterize other microbial groups (viruses and fungi) or to directly assess microbial metabolic functions or microbe-host interactions. If we are to characterize the functionality of the asthma microbiome and its interaction with the host during pathogenesis, high-resolution profiling genomic data from the host (transcriptomics) and microbial community (metatranscriptomics), and clinical and environmental data need to be generated and integrated using sophisticated analyses and computational tools.

Here we present a dual transcriptomic profiling analysis of the microbiomes of 14 asthmatic and non-asthmatic children for which host and microbial RNA-seq data has been collected. We then use several bioinformatic methods of high-throughput sequencing analysis to: 1) characterize the nasal microbiome functional diversity and human epithelial gene expression during health and disease in pediatric asthma; and 2) find associations between microbial shifts in function and composition and selected host genes (upstream regulators) involved on inflammatory and immune responses while accounting for clinical variation.

## Materials and Methods

### Ethics

All participants in this study were part of the AsthMaP2 (Asthma Severity Modifying Polymorphisms) Study. AsthMaP2 and the study presented here were approved by the Children's National Medical Center Institutional Review Board (Children's National IRB), which requires that consent is obtained and documented prior to conducting study procedures and collection of samples for research. Written consent was obtained from all independent participants or their legal guardians using the Children's National IRB approved informed consent documents.

### Samples and molecular analyses

Detailed information about the cohort and molecular methods can be found in Castro-Nallar et al. [12]. Briefly, nasal epithelial cells were collected from 8 children and adolescents (ages 6 to 17), both males (75%) and females (25%), with asthma and 6 healthy controls (ages 10 to 20; 83% males). Four of the asthmatics were using inhaled corticosteroids at the time of sampling. Total RNA was extracted using Trizol (Life Technologies) and chloroform reagents and the resulting lysate was used for affinity RNA purification in silica columns, following manufacturer’s instructions (Total RNA purification kit, Norgen Biotek, Canada). Total RNA was subjected to RiboZero ribosomal RNA reduction prior to library preparation using Illumina TruSeq Stranded Total RNA kit (San Diego, CA) and sequenced on a HiSeq 2500 platform at Boston University School of Medicine. An average of 41.4 million single-end 100 bp sequencing reads were generated per sample. Sequence data was deposited in the Sequence Read Archive and can be found under the BioProject [SRA: PRJNA255523].

### RNA sequence processing

Raw reads were preprocessed (QC) using PRINSEQ-lite 0.20.4 (trimming reads and bases < 25 PHRED, removing exact duplicates, reads with undetermined bases, and low complexity reads using Dust filter = 30) [25]. Filtered reads were aligned to the human genome (hg19) using Bowtie2 [26] (—very-sensitive-local). An average of 33.7 million reads per sample aligned to the human genome, while 1.8 million reads (microbial reads) were filtered off (S1 Table). Human reads were used for downstream analyses of host differential expression, while microbial reads were used for downstream analyses of microbial function.

### Microbial community composition and function

Taxonomic composition from microbial RNA data was inferred using PathoScope 2.0 [27–29]. A detailed characterization of the microbial composition of the microbiomes of asthmatic and non-asthmatic patients in this study is presented in Castro-Nallar et al. (2015). Taxonomic profiles identified in Castro-Nallar et al. (2015) will be used here to assess potential associations with the host gene expression during asthma. Metabolic functions of the microbiotas residing in asthmatic and non-asthmatic patients were inferred from metatranscriptome data by first aligning the microbial reads against the NCBI-NR protein reference database (as of Nov 2014) using DIAMOND [30]. Aligned gene reads (SAM files) with a bit-score of ≥50 were then loaded into MEGAN5 [31] and mapped to clusters of orthologous groups, functional roles, and metabolic pathways (categories 1–3) in the COG [32], SEED [33] and KEGG [34] databases, respectively. Only reads that mapped to bacterial genes were considered (0.96 to 1.8 million) and were aggregated across 1–3 categories for the three databases. Abundance differences between asthmatic and non-asthmatic children were compared in STAMP [35] using Welch’s test [36] or White’s non-parametric t-test [37]. Confidence intervals were estimated by the inverting Welch’s t-test and using a percentile bootstrapping method (10,000 replications), respectively. Results were depicted in post-hoc extended error bar plots (effect size filter: difference between proportions <1%). Differences between samples were also shown using principal component analysis in STAMP. False discovery rate (FDR) in multiple testing was controlled by using the Benjamini-Hochberg FDR [38] or Storey’s FDR [39] tests. Absolute counts were either converted to percentages and/or randomly subsampled (1,000 replicates) to the smallest count of any of the given samples.

### Host gene expression

Differential gene expression analysis was performed essentially as described by Trapnell et al. [40]. Briefly, human reads for each sample were aligned to the human reference genome (hg19) using TopHat2 [41]. Gene annotations from the UCSC knownGene database (genome.ucsc.edu/) were used to facilitate the mapping process. Transcripts present in each sample were assembled independently, then merged with the reference annotation using Cufflinks2 [40]. An average of 12.9 million reads per sample aligned to the human transcriptome. The merged transcriptome annotation was used to compute bias-corrected gene and transcript expression profiles for each sample using cuff quant [42]. We identified genes that were differentially expressed between asthmatics and healthy controls by treating samples as biological replicates; statistical significance and fold-change calculations were performed using cuffdiff [43]. Ingenuity Pathway Analysis (IPA) (www.ingenuity.com/products/ipa) software was then used for enrichment analyses of upstream regulators involved in inflammatory and immune responses during asthma. IPA predicts regulators that are likely to be activated or inhibited based on a given gene expression pattern and their activation z-score or *P* values.

### Associations between microbial profiles, host gene expression and clinical variables

To test for associations between microbiome functions and taxa and selected genes of interest from the host, while accounting for clinical variation, we applied the multivariate method implemented in MaAsLin (huttenhower.sph.harvard.edu/galaxy) [18]. MaAsLin performs boosted, additive general linear models between metadata (the predictors) and microbial abundance or function (the response). Boosting of metadata and selection of a model was performed per taxon. The following metadata were investigated in the analysis: asthmatic vs non-asthmatic and nasal epithelial gene expression of selected upstream regulators predicted by the IPA. We also controlled for age, gender and medication (corticosteroids) since these three variables may play a role in asthma phenotypic clustering [44] and microbial diversity [45]. All microbial data were arcsin-square root transformed [18]. Significant associations after multiple testing were considered below a *q* value threshold of 0.25 as recommended by the authors [18].

## Results

In this study, we assessed microbial and human gene expression and human-microbe associations in pediatric asthma while accounting for clinical information. In order to measure functional diversity in microbial communities and hosts, we subjected the RNA from microbiomes and human nasal epithelial cells of 8 asthmatic and 6 non-asthmatic (controls) patients to shotgun sequencing using the Illumina HiSeq platform. RNA reads from the microbes (metatranscriptome) and host (transcriptome) were *in silico* separated and analyzed using different bioinformatic pipelines.

### Host gene expression differs between asthmatic and non-asthmatic children

**Fig 1:**
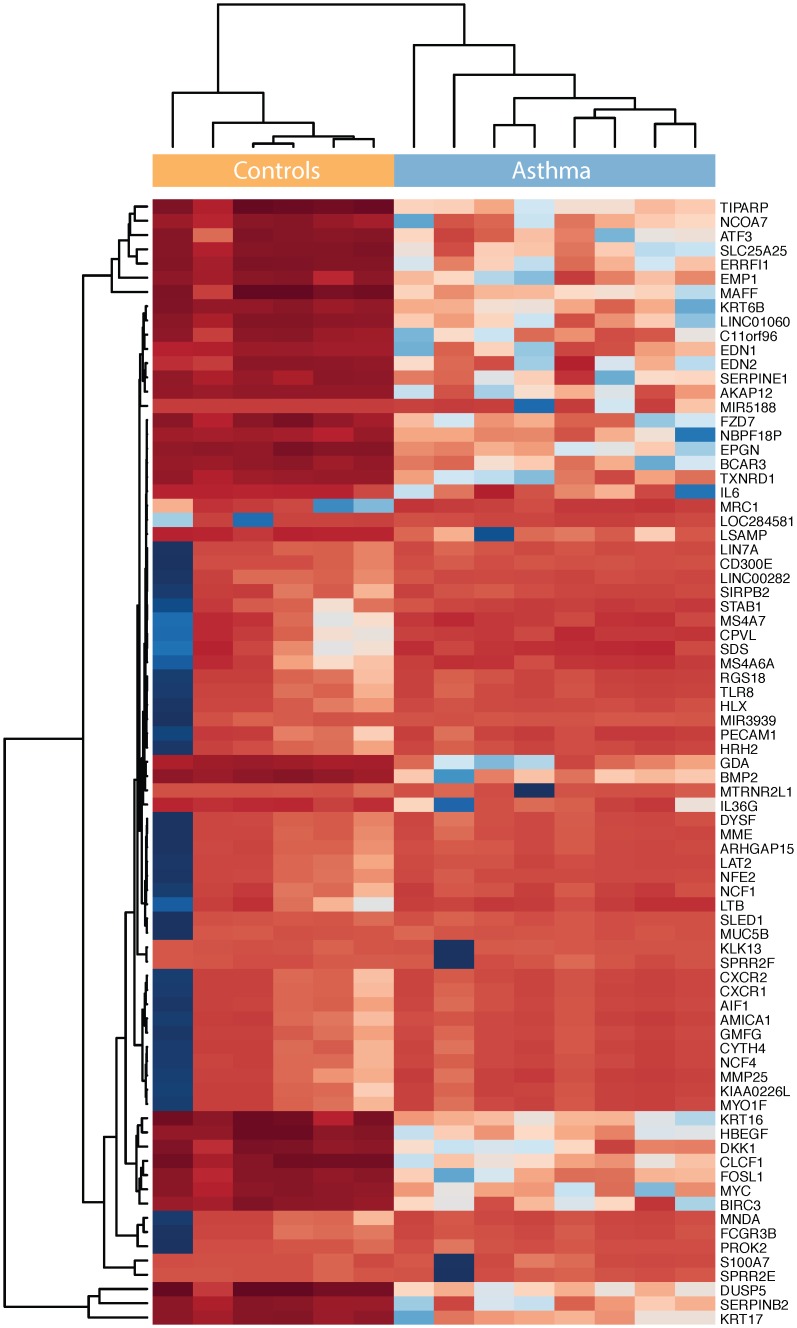
Heat map of 79 genes with a log-fold-change ≥3 between asthmatics and controls. Red to blue shows lower to higher proportions of sequences assigned to each sample.

**Fig 2:**
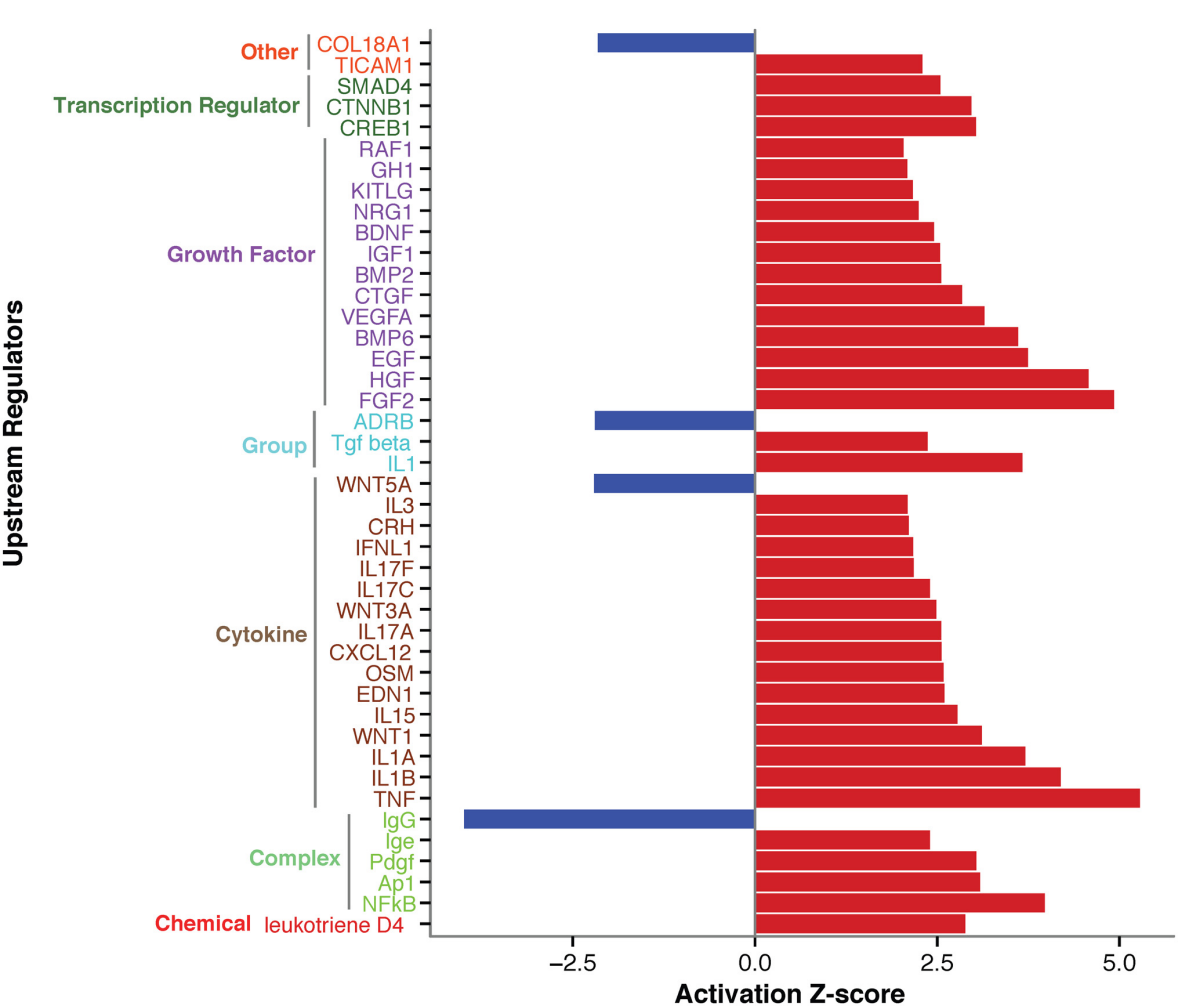
Ingenuity pathway enrichment analyses to detect upstream regulators associated with the inflammatory and immune responses during asthma. The activation *z*-score depicts the degree of activation or suppression of a given regulator.

Our transcriptomic analyses identified 499 (324 up-regulated and 175 down-regulated) core asthma genes differentially expressed (*P* < 0.003; log-fold-change ≥1.5) in the epithelial cells of asthmatic and non-asthmatic patients (S2 Table). Both were fully separated based on the abundances of the most differentially expressed (log-fold-change ≥3) 79 host genes (Fig 1). The core asthma signature (499 genes) was then loaded into IPA and enriched for genes connected to upstream regulator molecules related to inflammatory and immune responses during asthma [46–59]. Regulators were classified by group, including: complex, cytokine, group, growth factor, transcription regulator, chemical and other molecules. Four and 39 regulators were predicted to be inhibited (z-score ≤-2; *P* value range: 6.7E-05 to 2.9E-15) and activated (z-score ≥2; *P* value range: 1.9E-02 to 8.5E-50), respectively (Fig 2).

### Microbial metabolic functions differ between asthmatic and non-asthmatic children

**Fig 3:**
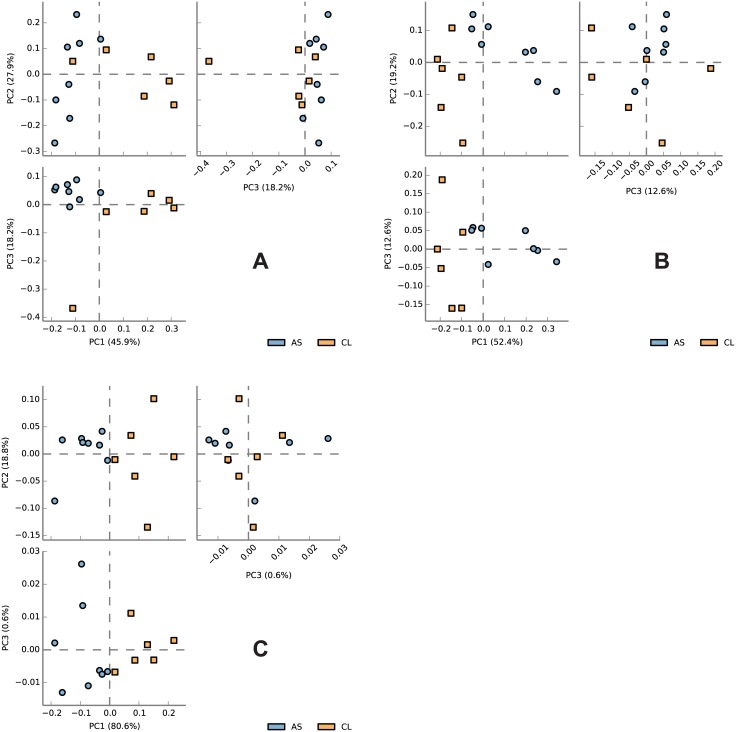
Principal Component Analysis (PCA) plot comparing functional profiles of asthmatics (AS) and controls (CL). PCA plots for COG category 2 (A), SEED category 2 (B) and KEGG category 3 (C).

**Fig 4:**
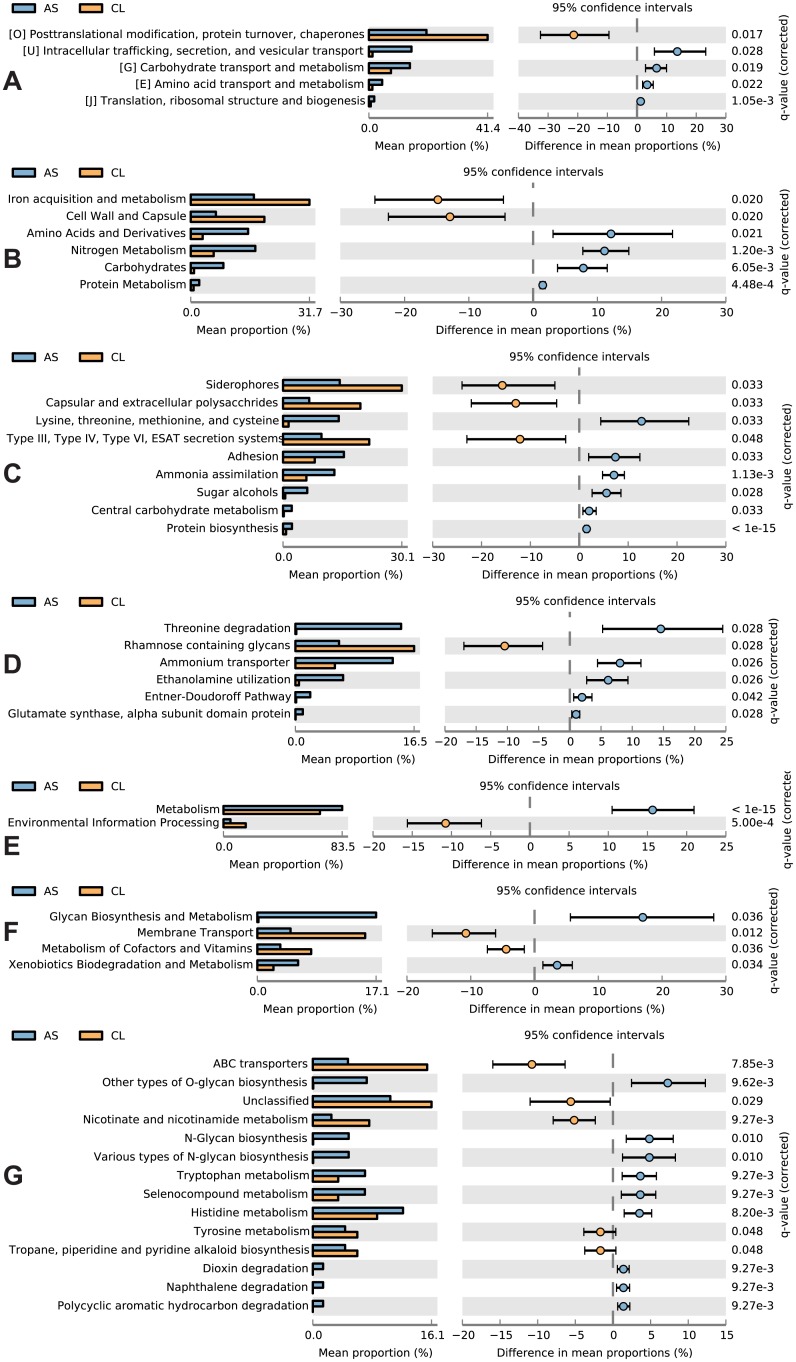
Extended error bar (EEB) plots showing functional properties differing significantly between asthmatics (AS) and controls (CL) with an effect size ≥1%. EEB plots for COG category 2 (A), SEED categories 1 to 3 (B–D) and KEGG categories 1 to 3 (E–G).

Here we performed an analysis of the metabolism of the bacterial communities residing in the nares of asthmatic and non-asthmatic children. Bacterial sequence reads (0.96–1.8 million per sample) in our RNA-seq experiment were assigned to 4 and 20 orthologous groups (COG; 285,672 reads); 25 to 171 functional roles (SEED; 347,061 reads) and 5 to 132 metabolic pathways (KEGG; 720,716 reads) in MEGAN5. These read numbers are higher or similar to those reported in other metatranscriptomic studies of the more diverse gut microbiota [20, 60]. Proportions of genes assigned to these functional properties varied between asthmatics and controls. Our principal component analysis confirmed that the metatranscriptomes of asthmatic children are relatively different from those of non-asthmatic children (Fig 3 and S1 Fig). Moreover, some of the component pairs in the analyses clustered the asthmatic microbiomes together, suggesting that they are more similar to each other in their functionality than the microbiomes of the non-asthmatic controls (Fig 3A; PC1 vs PC3). Multiple functional properties were differentially expressed during health and disease across different functional categories 1–3, but after FDR correction (*q*-value < 0.05), only 5 orthologous groups (COG), 6–9 functional roles (SEED), and 2–14 metabolic pathways (KEGG) remained significant (Fig 4). These differences were consistent within groups and showed and effect size of at least 1%. All these properties remained significant when accounting for clinical variation in age, gender and medication in our multivariate analysis (MaAsLin). There was considerable overlap (i.e., congruence) between analyses, with most of the selected (i.e., significant) properties involving basic metabolism (e.g., biosynthesis and degradation) and metabolite transport routes. Protein metabolism showed major perturbations, with several genes involved in the metabolism, biosynthesis, and degradation of amino acids and derivatives (threonine, histidine, tryptophan, tyrosine, phenylalanine) varying between asthmatics and controls. Nitrogen metabolism also differed between asthmatic and non-asthmatic microbiomes, with ammonium transporter and glutamate synthase overexpressed in the former. Asthma was also associated with increased abundance of genes related to central carbohydrate metabolism (Entner-Doudoroff Pathway) and sugar alcohols (ethanolamine utilization). Similarly, genes involved in xenobiotic biodegradation (e.g., dioxin, naphtalene) and glycan biosynthesis were also more abundant in asthmatic microbiomes. Finally, within the virulence, disease and defense SEED subsystems (Fig 4C), adhesion had more sequence hits in asthmatics, while type III, IV and VI and ESAT secretion systems had less. At the most exclusive level, 4/5, 5/6 and 9/14 of the significantly different orthologous groups (COG), functional roles (SEED), and metabolic pathways (KEGG), respectively, were enhanced in asthmatic samples, which strongly suggests that they may be important for stability of disease-associated populations and likely contribute to the disease process.

### Microbial metabolic functions and composition are associated with upstream regulators involved in immune and inflammatory responses during asthma

**Fig 5:**
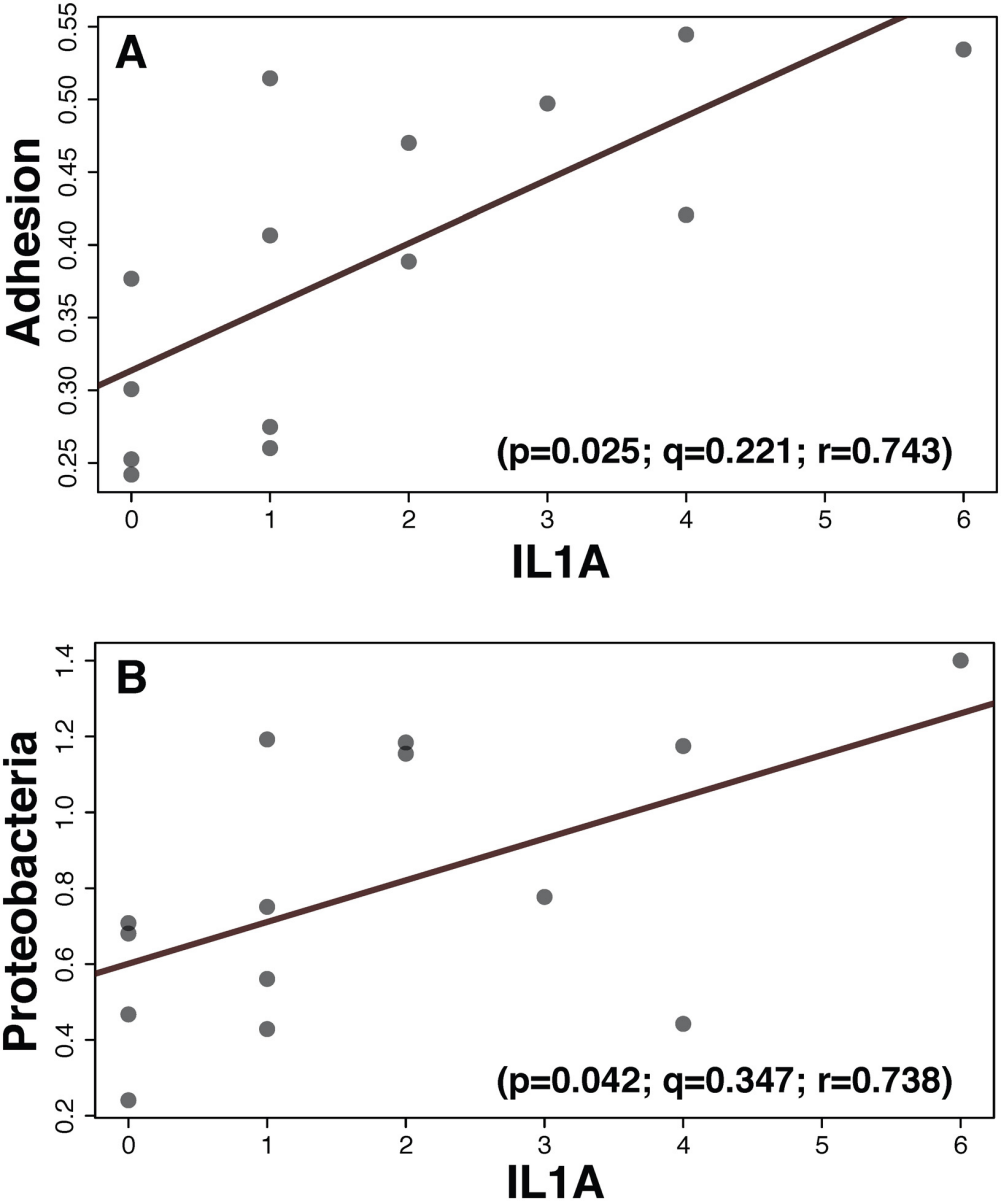
MaAsLin scattergrams showing associations between the microbial functional property of Adhesion (A) and proportions of Proteobacteria (B) and change in expression of the host gene regulator *IL1A*. *P* and *q* value tests for significance and correlation coefficients are shown.

For the purpose of uncovering potential microbe-host interactions in the nasal epithelial cells, we used a multivariate approach (MaAsLin) to examine the multifactorial structure between taxonomic and functional characteristics of the microbiota and selected host transcriptome upstream regulators. Given the limited number of samples in our study (14 patients) and the high number of variables in our data and metadata (some also continuous), and to maintain statistical power in our association analysis while testing biologically meaningful interactions (i.e., microbiome shifts influence immune response), we restricted the number of host upstream regulators, microbial groups and functions tested in our host-microbe multivariate analysis, while controlling for phenotype, age, gender and medication. Of the predicted 43 upstream regulators involved in immune and inflammatory responses (Fig 2), we tested the 8 regulators (IL1A, EDN1, TGFA, VEGFA, BMP2, COL18A1, TICAM1 and NFKB1) also present in our host gene expression analysis. Three of those genes, *IL1A* (interleukin 1 alpha), *EDN1* (endothelin 1) and *NFKB1* (nuclear factor of kappa light polypeptide gene enhancer in B-cells 1) were also found to be activated in pharyngeal and lung epithelial cell cultures in response to infection with *Moraxella catarrhalis* [61]. Accordingly, to constrain the number of microbial functions and taxa in the MaAsLin analysis, we only tested categories 2 in our orthologous groups (COG), functional roles (SEED), and metabolic pathways (KEGG) and the most inclusive microbial taxonomic profile (Phylum) in Castro-Nallar et al. (2015). Moreover, functional properties and Phyla not present in at least 50% of the asthmatics or controls were excluded. Our multivariate analysis detected one significant association (*P* = 0.025) between *IL1A* and the microbial functional property of adhesion (SEED) (Fig 5A). Similarly, the bacterial Phylum Proteobacteria was also significantly associated (*P* = 0.042) to *IL1A*, but this association did not remain significant after FDR correction (Fig 5B). These associations are without the influence of the other metadata (phenotype, sex, age and medication) in the study.

## Discussion

### Microbiome functional diversity

Several studies have shown that microbial infection is a contributing factor to asthma and that microbial communities in the respiratory airways vary between health and disease (see reviews in [5, 6–9]). Despite these findings, it is unknown how specific activities of different members of the community impact disease. This study represents the first metabolic reconstruction of the microbial populations residing in nasal nares and identifies numerous functions and pathways associated with healthy and diseased asthmatic children.

Our functional analyses of the most exclusive categories analyzed here identified 5/20 (25%) orthologous groups (COG), 6/171 (3.5%) roles (SEED) and 14/132 (10.6%) metabolic pathways (KEGG) differentially expressed (*q* < 0.05) in asthmatic and non-asthmatic children. These same analyses applied to taxonomic profiles in Castro-Nallar et al. [12] only detected 1/174 (0.6%) families (Moraxellacea) and 1/797 bacterial species (*Moraxella catarrhalis*) significantly different in their proportions between asthmatics and controls. *M*. *catarrhalis* is a pathogen that has been associated with childhood asthma [11, 62]. Hence, this comparison suggests that metabolic activities are more perturbed in asthma than are organismal abundances, as previously indicated in inflammatory bowel disease [18]. Moreover, *Moraxella* accounted for a great proportion of the microbial transcripts; therefore, the differences in metabolism seen here between asthmatic and non-asthmatic subjects are, to a large extent, due to differences in expression of *Moraxella* genes. This constitutes an exemplar case of how changes in the taxonomic composition of one bacterium during disease can lead to major modifications in the metabolic functions of the microbial community.

Most microbiome functional analyses, like the one presented here, aim to describe and compare healthy and diseased microbiotas. Further insight into the microbiome dysfunction in asthma can be gained from prior knowledge of these metabolic pathways in known asthma pathogens and from comparisons with other microbiome studies. Very little information, however, has been accumulated in this regard with most research focused on the gut and oral microbiota [16–18, 20, 22, 63]. Other studies, however, [61, 64], have used cell cultures to look at the gene expression of *M*. *catarrhalis* genes in pharyngeal and lung epithelial cells and, like here, have shown increased expression of genes involved in central metabolism, transport proteins and adhesion (virulence) functions. Similar to these studies, our functional analyses in asthmatic children revealed general perturbations of the amino acid, carbohydrate and vitamin metabolism and transport, xenoniotic degradation and virulence. This reinforces the idea of a “common mucosal response” [65], and suggests that either these functions play a common role in gastrointestinal-lung human diseases or that oral and gut microbial communities share members or exchange metabolites across mucosae [66]. Our results represent the first step towards the functional investigation of asthma microbiotas. As sequencing costs continue to fall, new metatranscriptomes combined with proteomic and metabolomic data will generate insights in our understanding of the microbial functionality and which of those functions are most strongly affecting the host during disease.

### Immune and inflammatory responses during asthma

Our transcriptomic analyses identified a core of 499 genes differentially expressed in the epithelial cells of asthmatic and non-asthmatic patients. Using that set of genes, IPA enrichment predicted 39 and 4 upregulators to be activated or inhibited, respectively. These upregulators have been associated with cell and tissue proliferation and reconstruction, cell-mediated and humoral immunity responses and inflammation during asthma (as indicated by experimentally observed relationships and references in IPA and in [46–59]). Our analysis shows that most of these cell and tissue processes are activated (z-score ≥2) in asthmatics; as seen in human and mouse models assessing airway remodeling in asthma [46, 47, 51, 53, 55–58, 67–71]. Untangling the metabolic pathways and networks displayed by these regulators and examining in detail the functions of the genes connected to them is beyond the scope of this study. Our ultimate goal here was to illustrate the potential of dual transcriptomic profiling and find candidate genes in our host transcriptomic data that could interact with the microbiome (see next section).

### Microbe-host interactions

Microbe-host interactions are expected to play a significant role in the development of multifactorial diseases like asthma. Here we present a dual profiling analysis of host and microbial genes to assess such interactions in relation to immune and inflammatory responses. Our multivariate analysis showed a significant association between the host gene *IL1A* and the virulence characteristic of adhesion of the microbiome—an increase of adhesion is positively associated with an upregulation of *IL1A*. The protein encoded by this gene is a member of the interleukin 1 cytokine family. This cytokine is a pleiotropic cytokine involved in various immune responses, inflammatory processes, and hematopoiesis. This association is also supported by previous studies linking up-regulation of *IL1A* and other interleukins to epithelial infection by *M*. *catarrhalis* [61, 64]; moreover, *IL1A* has also been correlated to virulence characteristics of the gut microbiome in infants [20]. Similarly, our multivariate analyses also suggested that an increase in Proteobacteria is potentially related to specific changes in the expression of *IL1A*. Significant increases in Proteobacteria have been associated with pediatric asthma [3, 11, 12, 62, 72]. Our analysis, hence, reveals that functional and compositional changes in the microbiome contribute to or modulate host mucosal inflammation and immune response during asthma.

Our study describes a pipeline to identify interactions between key host regulators (as predicted by ingenuity pathway enrichment and reconstruction) and microbial characteristics. Other approaches based on linear models and canonical correlation analysis have been also described to investigate similar associations [20, 73, 74]. Given the multifactorial nature of respiratory diseases and the high number and diversity of the potential variables (continuous, discrete, Boolean) under study, comprehensive microbe-host multivariable analyses require large sample sizes. Future high-throughput sequencing platforms will eventually reduce RNA sequencing costs to make the study of large cohorts affordable. But given the data currently at hand, multivariate analyses like those presented here could be readily applied to 16S metagenomic and host microarray gene expression profiles under different clinical and environmental settings [e.g., 18, 73, 75].

## Conclusions

Our study demonstrates how metagenomic data from the same RNA-seq experiment can be processed with powerful bioinformatic approaches to simultaneously characterize human and microbiome functional diversity and infer microbe-host interactions. Our analyses show 1) that the metabolism of both the bacterial communities in the nasal cavity and the host nasal epithelial cells differ between asthmatic and non-asthmatic children, and 2) that microbiome variation in function and composition may impact host metabolic regulation during immune and inflammatory responses. We hope our study inspires future research on respiratory airways driven to determine the contributions and interactions of microbes, host, and environment to and during health and disease, and to eventually elucidate whether microbiome changes are the cause or the consequence of those diseases.

## Supporting Information

S1 Fig Principal Component Analysis (PCA) plot comparing functional profiles of asthmatics (AS) and controls (CL). PCA plots for COG category 1 (A), SEED categories 1 and 3 (B, C) and KEGG categories 1 and 2 (D, E).
(TIFF)

S1 Table Host gene expression analysis. Core asthma genes differentially expressed (*P* < 0.003; log-fold-change ≥1.5) in the epithelial cells of asthmatic (P001-P008) and non-asthmatic (P009-P014) patients.
(XLSX)

S2 Table Mapped reads. Raw and filtered (QC) reads mapped to the human genome and transcriptome, microbial reads and bacterial reads mapped to the NCBI-NR database with a bit-score ≥50.
(XLSX)
